# Syntenin controls migration, growth, proliferation, and cell cycle progression in cancer cells

**DOI:** 10.3389/fphar.2015.00241

**Published:** 2015-10-21

**Authors:** Rudra Kashyap, Bart Roucourt, Frederique Lembo, Joanna Fares, Ane Marcos Carcavilla, Audrey Restouin, Pascale Zimmermann, Rania Ghossoub

**Affiliations:** ^1^Laboratory for Signal Integration in Cell Fate Decision, Department of Human Genetics, KU LeuvenLeuven, Belgium; ^2^Centre de Recherche en Cancérologie de Marseille, Aix-Marseille UniversitéMarseille, France; ^3^Inserm U1068, Institut Paoli-CalmettesMarseille, France; ^4^Centre National de la Recherche Scientifique, UMR7258Marseille, France

**Keywords:** syntenin, PDZ proteins, syndecan, cancer cell migration, cancer cell growth, cancer cell proliferation, cell cycle

## Abstract

The scaffold protein syntenin abounds during fetal life where it is important for developmental movements. In human adulthood, syntenin gain-of-function is increasingly associated with various cancers and poor prognosis. Depending on the cancer model analyzed, syntenin affects various signaling pathways. We previously have shown that syntenin allows syndecan heparan sulfate proteoglycans to escape degradation. This indicates that syntenin has the potential to support sustained signaling of a plethora of growth factors and adhesion molecules. Here, we aim to clarify the impact of syntenin loss-of-function on cancer cell migration, growth, and proliferation, using cells from various cancer types and syntenin shRNA and siRNA silencing approaches. We observed decreased migration, growth, and proliferation of the mouse melanoma cell line B16F10, the human colon cancer cell line HT29 and the human breast cancer cell line MCF7. We further documented that syntenin controls the presence of active β1 integrin at the cell membrane and G1/S cell cycle transition as well as the expression levels of CDK4, Cyclin D2, and Retinoblastoma proteins. These data confirm that syntenin supports the migration and growth of tumor cells, independently of their origin, and further highlight the attractiveness of syntenin as potential therapeutic target.

## Introduction

Syntenin is strongly expressed during human fetal life and at relatively low levels in adult tissues (Zimmermann et al., 2001). Loss-of-function studies in Xenopus and zebrafish indicated that syntenin plays an important role in early developmental movements by controlling the non-canonical Wnt-signaling pathway (Luyten et al., 2008; Lambaerts et al., 2012), among others. Loss of syntenin function in mice has comparatively little effects. Indeed, Tamura et al. (2015) recently reported a mild phenotype in intestinal homeostasis.

Syntenin is detectable in adult human tissues, but an increasing number of independent studies indicate that syntenin is overexpressed in various patient tumor samples. Syntenin gain-of-function was first described in metastatic melanoma (Helmke et al., 2004), and more recently in breast cancer (Qian et al., 2013; Yang et al., 2013) and in multiple neuroepithelial tumors (Kegelman et al., 2014) suggesting that syntenin could be a tumor marker. For example, in breast cancer patients, the correlation between syntenin expression, tumor size, lymph node status, and recurrence appears statistically significant (Yang et al., 2013).

In cellular models, by *in vitro* but also *in vivo* approaches with xenografts, several studies have shown that elevated syntenin expression is particularly relevant for invasion and metastasis (Koo et al., 2002; Boukerche et al., 2005; Das et al., 2013; Liu et al., 2014). Depending on the cellular context, syntenin has been associated with the activation of various signaling pathways, including SRC/p38MAPK/NFkB in human melanoma (Boukerche et al., 2005, 2007, 2008, 2010), in human glioblastoma multiform (GBM) (Kegelman et al., 2014), and in head and neck squamous cell carcinoma angiogenesis (Oyesanya et al., 2014), integrin β1/ERK1/2 in human breast cancer cells (Yang et al., 2013), EGFR/Akt/PI3K in urothelial cell carcinoma (Dasgupta et al., 2013), HIF-1α/IGFBP-2 in human melanoma angiogenesis (Das et al., 2013), and STAT3/PI3K/CTNNB1 in head and neck squamous cell carcinoma angiogenesis (Oyesanya et al., 2014).

Syntenin is a scaffold protein containing two Post synaptic density-95, Disc-large tumor suppressor and Zonula occludens-1 (PDZ) domains that we originally identified as an intracellular adaptor for the syndecan family of heparan sulfate (HS) proteoglycans (Grootjans et al., 1997). HS proteoglycans are highly abundant in adherent cells and their HS chains have numerous ligands, including various morphogens, adhesion molecules, and growth factors, such as Wnts, fibronectin and FGFs, whose deregulated signaling is involved in cancer development and progression (Fuster and Esko, 2005). HS plays an important role in the docking of these factors to cognate signaling receptors and can connect and regulate many signaling systems in a cell-type and cell-context dependent manner. Besides interacting with syndecans, the PDZ domains of syntenin can also directly interact with various membrane proteins and receptors (Beekman and Coffer, 2008), including Frizzled Wnt receptors that can rely on syndecans for their functions (Luyten et al., 2008). In structure–function studies, we demonstrated that syntenin allows syndecans and associated molecules to escape degradation by promoting their recycling to the plasma membrane (Zimmermann et al., 2005) or their secretion as exosomal cargo (Baietti et al., 2012; Ghossoub et al., 2014; Friand et al., 2015; Roucourt et al., 2015). These studies are entirely consistent with the observation that syntenin can boost various signaling pathways when overexpressed in cancer cells. The functional versatility of syndecans also explains that syntenin gain-of-function can support various signaling pathways and that specific effects can be cell-type dependent.

As a starting point to evaluate the potential benefit of anti-syntenin drugs, we here aimed to document and compare the impact of syntenin loss-of-function on the migration, invasion, growth, and proliferation of various model cancer cell lines.

## Materials and Methods

### Cell Culture and Transient Transfections

HT29, MCF7, and B16F10 cell lines were purchased from the American Type Culture Collection (Manassas, VA, USA). HT29 cells were grown in McCoy’s medium (Thermofisher Scientific), MCF7 cells in DMEM-F12 medium (Thermofisher Scientific), and B16F10 cells in DMEM medium (Thermofisher Scientific). Media were supplemented with 10% fetal bovine serum (FBS) (Thermofisher Scientific) and cells were incubated at 37°C under 5% CO2. For transient expressions, cells were plated 24 h earlier at a density of 1 × 10^5^ cells per well in six well plates (BD Falcon) with 2 ml medium. 4 μl of Fugene HD reagent (Roche Applied Sciences) were added to 200 μl Opti-MEM solution (Thermofisher Scientific) and 1 μg plasmid DNA. The mixture was incubated for 20 min at room temperature before being added to the cells. For RNAi experiments, cells at a confluence of 50% were transfected with 20 nM RNAi using Lipofectamine RNAiMAX reagent (Life technologies, USA). Cells were analyzed after indicated time.

### Expression Vectors and Reagents

RNAis targeting Syntenin and the non-targeting control RNAi (si Ctrl) were purchased from GE healthcare Dharmacon Inc (Human syntenin (5′-GCAAGACCUUCCAGUAUAA-3′), Mouse syntenin smartpool (M-043821-01)). For rescue experiments, syntenin cDNA was cloned in pcDNA3.1/Zeo(+) (Thermofisher Scientific) and mutated by directed mutagenesis on three nucleotides in the sequence targeted by the siRNA (CCTTCCAGT mutated to CCGTCGAGC).

Empty vector, control shRNA and the 29 mer human and mouse shRNA sequences cloned in pGFP-V-RS vector were purchased from Origene [control shRNA GCACTACCAGAGCTAACTCAGATAGTACT (TR30013), Human syntenin shRNA 2 (GCCTAATGGACCACACCATTCCTGAGGTT (TG309594B/GI338370), shRNA 3 (GTGGCTCCTGTAACTGGTAATGATGTTGG (TG309594C/GI338371), Mouse shRNA (TCAGGCTCAAACTGCTTATTCTGCCAATC (TG512166A/GI574570)].

### Statistical Analysis

Statistical Analysis was performed using the standard two-tailed Student *t*-test, and ^∗^*P* < 0.05, ^∗∗^*P* < 0.01, ^∗∗∗^*P* < 0.001 were considered statistically significant. Metamorph, Image J and ColonyDoc-It acquired data were processed with GraphPad Prism software.

### Preparation of Cancer Cell Lines Depleted in Syntenin

Empty vector, control shRNA, and syntenin shRNAs expression vectors for mouse and human syntenin were transfected in phoenix packaging cells [ecotropic for Mouse and amphotropic for human cells (Life Technologies)]. Viral supernatants were harvested after 24–48 h intervals and used to infect cells for 48 h. Stable populations were selected for 10 days using optimal concentrations of puromycin as tested firstly on non-transduced cells (minimal concentration for death).

### Generation of Syntenin Antibody

For the immunization, two rabbits were injected with two peptides, respectively, NEAEICESMPMVSGA and PSIM KSLMDHTIPEV, corresponding to sequences found in the N-Terminal and the C-terminal part of mouse syntenin-1 (Eurogentec). Crude sera from the two rabbits were pooled. Anti-syntenin antibodies were purified using the C-terminal peptide antigen with a carboxy terminal cysteine and a thiopropyl sepharose 6B column (Amersham). The unbound proteins were washed with sodium phosphate buffer. Antibodies were eluted with 0.1 mM glycine pH 2.5. Purified antibodies were immediately brought back to pH 7.5 using a solution of 1.5 mM TRIS-HCl pH 8.5. Antibodies were dialyzed overnight against PBS containing 0.01% sodium azide, 0.1% BSA, pH 7.5 overnight at 4°C and aliquots were stored at -20°C. The purified antibodies were shown to recognize mouse and human syntenin-1 using recombinant proteins. Their reactivity was lost on syntenin-1 CRISPR-Cas9 knock-out human cells and fibroblasts originating from syntenin-1 knock-out mice, attesting for their specificity.

### Western Blotting

Cells were plated in 10 cm diameter dishes. After 48 h, cell lysates were fractionated by SDS-PAGE and transferred to nitrocellulose membranes (GE Healthcare Life sciences). The membranes were blocked with 5% fat free milk and incubated with primary antibodies against Syntenin (homemade, see generation of syntenin antibody paragraph), β Actin (Santa Cruz, sc-69879), Tubulin (Sigma–Aldrich, USA), Cyclin D2 (Santa Cruz, sc-593), CDK4 (Cell signaling #2906), Retinoblastoma (BD Biosciences # 554136), and HRP-conjugated secondary antibodies. The membranes were washed with PBS/0.1% Tween buffer and antibody binding was revealed using enhanced chemiluminesescence (ECL) reagent (Thermo Scientific) according to the recommendations of the manufacturer. Signals were detected on photographic films (GE healthcare).

### Wound Healing Assay

Cells were treated with 1 ng/ml mitomycin (Sigma M4287) overnight to inhibit cell division. Treated cells were plated in Ibidi culture-inserts (Ibidi Cat-80206) for 12 h to reach 90–95% of confluence. A wound was created by removing inserts from the dishes. Medium was refreshed to remove dead cells and the cells were observed under an inverted light microscope (Leica SP2) equipped with a camera. Images were taken by MetaMorph software every 10 min for 24 h_._ Measurements of cell velocity were calculated using the MetaMorph software.

### Transwell Migration Assay

Cells were serum starved and treated with 1 ng/ml of mitomycin (Sigma M4287) for 12 h to inhibit cell division. Upper chambers (8 μm pores, Becton Dickinson, USA) cell chambers were placed in 24-well format transwell plates. Starved cells were plated in assay media (200 μl) (20 000 cells/well in the upper chamber). Lower chambers were pre-coated for 2 h with rat tail collagen I (Roche). To initiate migration, media containing 10% serum (600 μl) was placed in the lower chamber as attractant. After 48 h of incubation, cells were washed with PBS, fixed with 4% paraformaldehyde for 10 min, and stained with 0.5% crystal violet for 15 min at room temperature. Inserts were washed with PBS and air dried for 15 min. Cells on the upper side (non-migrating) of the filter were removed by swabbing with cotton wool. Migrating cells were imaged on a Zeiss Axioskop 2 fluorescence microscope using a 10x objective (Zeiss, NA 0.3) and a Spot RT/SE CCD camera (Diagnostics) in mosaic format. Cells were counted using ImageJ software cell counter.

### Plate Colony Formation Assay

Cells were counted using Malassez slides and seeded at 1000 cells per 6 cm diameter dish in the usual media for each cell line (see above). The culture media was changed every 5 days. After incubation for 14 days, cells were washed with PBS, fixed with 4% paraformaldehyde for 10 min, and stained with 0.5% crystal violet for 15 min at room temperature. Pictures were taken; visible colonies were counted using an automatic colony counter (UVP, ColonyDoc-It^TM^ Imaging Station).

### Soft Agar Colony Formation Assay

Agarose was melted in water and mixed with medium pre-incubated at 37°C at end-concentrations of 0.6 or 0.36%. A lower layer (0.6% agarose) was deposited in each well of a 96 well plates, except for the first and last rows and columns that were filled with PBS to avoid drying. Agarose was allowed to cool for 15 min before addition of the upper layer containing 2000 cells and 0.36% agarose. Plates were incubated at 37°C for 21 days in a 5% CO2 incubator and observed under a microscope at 24 h intervals. At the end of the experiments, cells were tested for their ATP content, an indicator of their metabolic activity, using the Promega kit G7570. In this assay, ATP is used as a co-factor for a luciferase reaction. Signals were analyzed by measuring luminescence with a LUMI starluminometer (BMG Labtech).

### Cell Proliferation

1 × 10^5^ cells were plated in 6 cm diameter dishes in six well culture plates. At each time point, a plate from each cell line was trypsinised, cells were collected in 2 ml of media and stained with Trypan blue. Cell counting was performed with Countess^®^ Cell Counting Chamber Slides (Thermo fisher C10228).

### Immunofluorescence Staining and Confocal Microscopy

Cells were cultured on glass coverslips, fixed with 4% PFA for 15 min, washed in PBS and then incubated with Integrin β1 antibody (BD 553715) in PBS containing 0.3% BSA and 0.05% saponin. Coverslips were mounted in DABCO/Mowiol and observed with a Zeiss Meta confocal microscope (LSM 510 META, Zeiss, France) with a UV laser and a 40× objective. Confocal images were analyzed and mounted using Photoshop (Adobe, San Jose, CA, USA) software.

### Cell Cycle Analysis

MCF7 cells were transfected with non-targeting and Syntenin siRNAs in 10% serum. After 24 h cells were synchronized in G_1_ phase by serum starvation for 24 h. After synchronization, cell cycle entry was recovered in fresh media and cells were analyzed at different time points. Therefore, cells were washed with cold PBS and fixed in 70% ethanol at 4°C for 30 min. After fixation cells were stained with a 20 μg/mL propidium iodide solution (PBS containing 40 μg/mL RNase A) for 30 min at 37°C. Cell cycle distribution in G_1_, S, and G_2_/M phases was analyzed on the BD LSRFORTESSA (BD Biosciences) flow cytometer. The percentage of cells in each cell cycle phase was analyzed by FACS Diva software.

## Results

### Generation and Validation of Various Cancer Cell Lines Depleted in Syntenin

**FIGURE 1:**
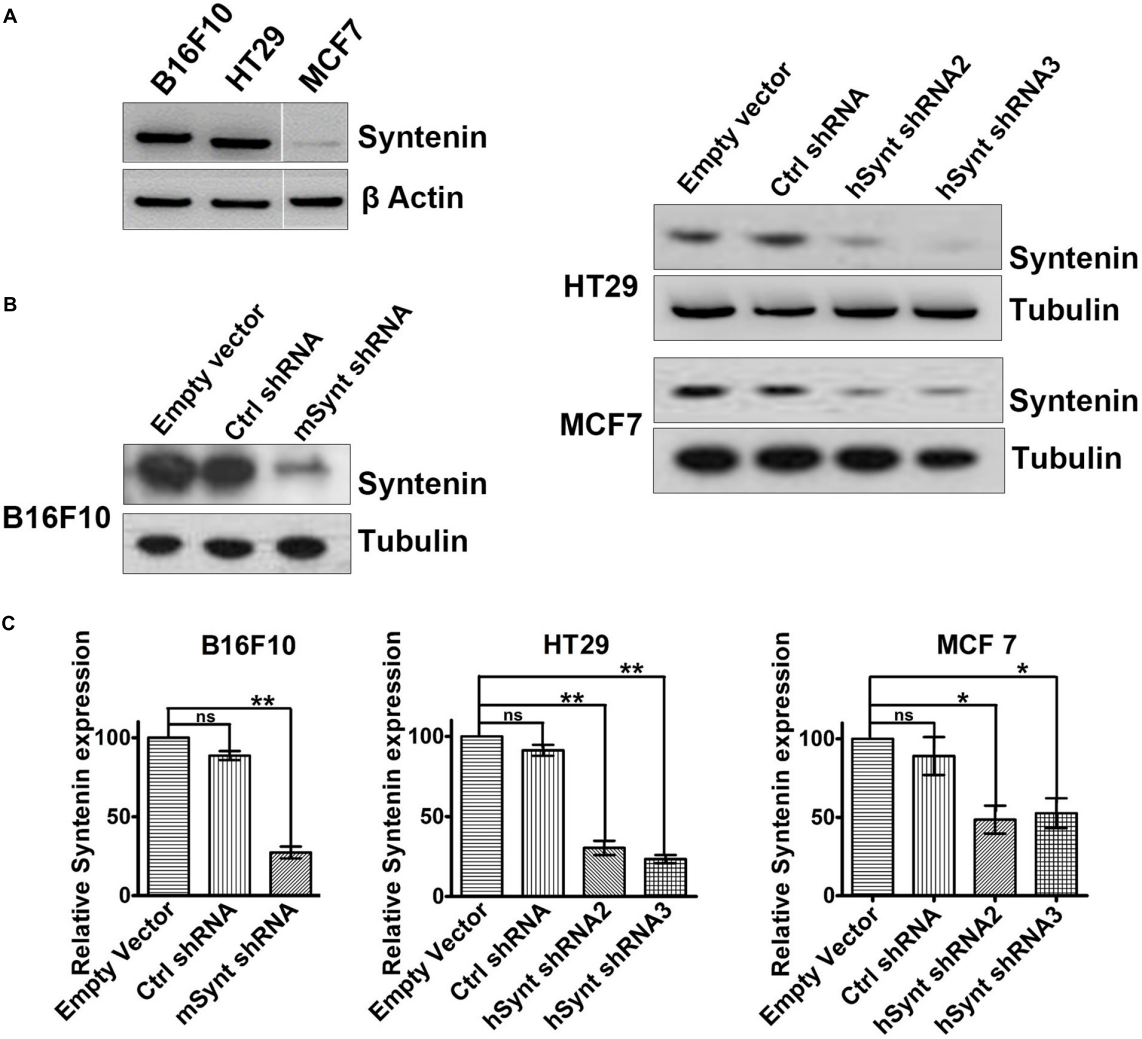
**Generation of cells with stable syntenin knockdown. (A)** Western blots illustrating Syntenin basal expression in B16F10, HT29, and MCF7 cells, data are from the same blot. Actin was used as a loading control. **(B)** Western blots illustrating Syntenin expression levels after viral transduction with various constructs, as indicated. One small hairpin RNA (mSynt shRNA) and two different shRNA molecules (hSynt shRNA2 and hSynt shRNA3) were used to efficiently knockdown syntenin expression in mouse (B16F10) and human cells (HT29 and MCF7), respectively. Empty vector and ctrl shRNA were used as controls. Tubulin was used as a loading control. **(C)** Bar graphs indicate the average levels of syntenin expression, expressed relative to levels in empty vector transduced cells (taken as 100%). *n* = 3, bars represent mean value ± SD, n.s., non-significant, ^∗^*P* < 0.05, ^∗∗^*P* < 0.01 (Student’s *t*-test).

To evaluate the impact of syntenin loss-of-function in cancer cells, we selected three different models: mouse melanoma B16F10 (mixture of spindle-shaped and epithelial-like cells, mutated for p53, RAS wild-type, highly metastatic and invasive), Human colorectal adenocarcinoma HT29 (epithelial cells, mutated for p53, RAS wild-type, highly metastatic, and invasive) and Human breast adenocarcinoma MCF7 (epithelial cells, ER-positive, p53, and RAS wild-type, non-metastatic, poorly invasive). B16F10 and HT29 cells, express higher levels of syntenin in comparison to MCF7 cells (**Figure 1A**). To generate cell populations stably depleted for syntenin, we used retroviral-mediated gene transduction of small hairpin RNAs (shRNA). One shRNA molecule (shRNA) and two different shRNA molecules (shRNA2 and 3) were used to efficiently knockdown syntenin expression in mouse and human cells, respectively (**Figure 1B**). After selection, the levels of syntenin were assessed by Western blot. The control shRNA, used as control, did not significantly affect syntenin expression when compared to empty vector transduced cells. shRNA targeting syntenin induced a significant decrease in syntenin expression in B16F10 cells (down by 80% compared to control levels), in HT29 cells (down by 70 and 75% compared to control levels for the two different shRNA, respectively) and in MCF7 cells (down by 60 and 65% compared to control levels for the two different shRNA, respectively) (**Figure 1C**).

### Syntenin Loss-of-function Impairs Cell Migration

**FIGURE 2:**
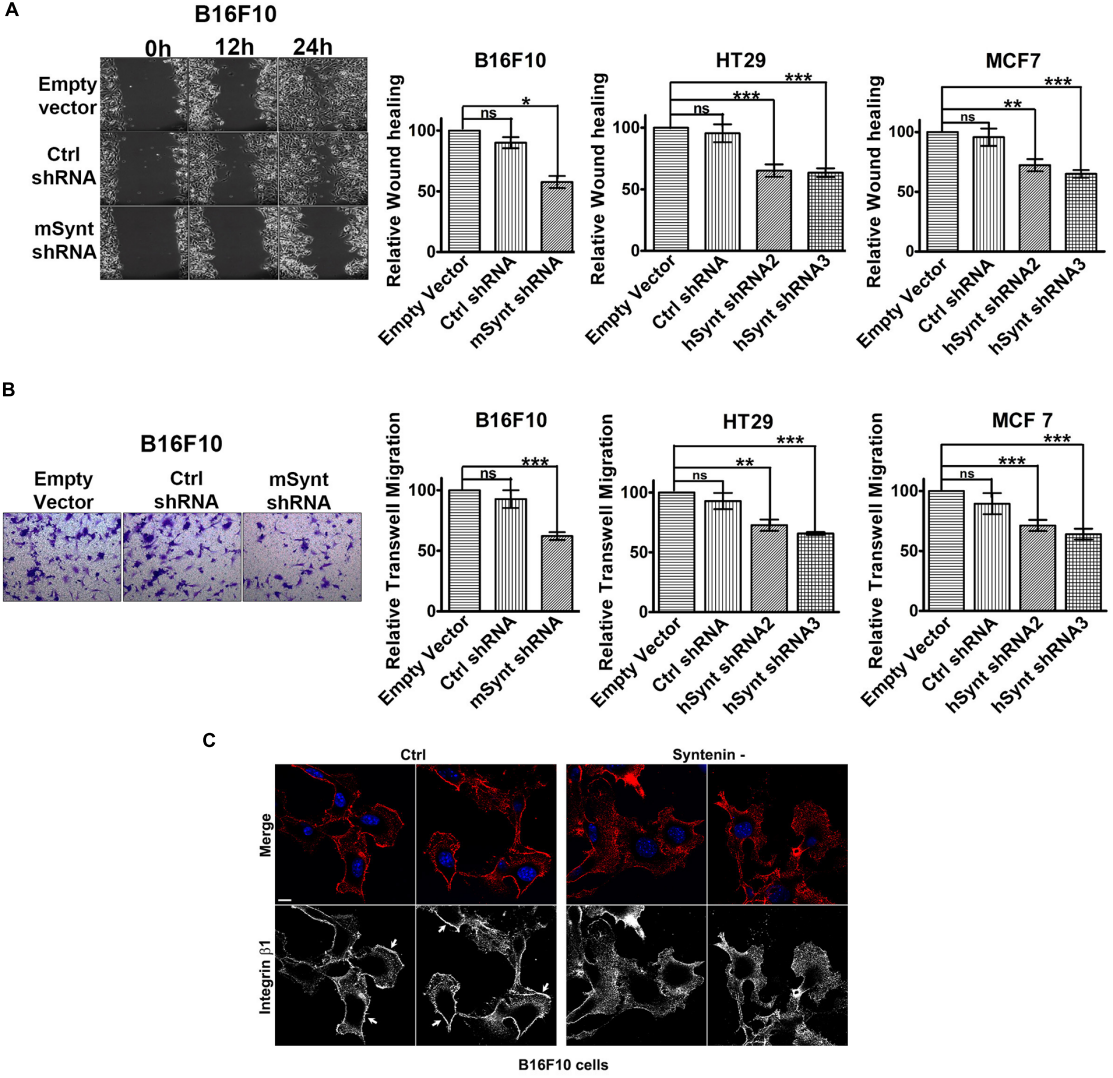
**Syntenin loss-of-function reduces the migration of mouse and human cancer cells. (A)** Left: Phase-contrast micrographs illustrating migration by wound healing of B16F10 cells monolayers transduced with empty vector, control, or syntenin shRNA. Images were taken at different time points after wounding, as indicated. Right: Bar graphs indicate gap closures relative to closure in empty vector transduced cells (taken as 100%). n = 3, bars represent mean value ± SD, n.s., non-significant, ^∗^*P* < 0.05, ^∗∗^*P* < 0.01, ^∗∗∗^*P* < 0.001 (Student’s *t*-test). **(B)** Left: Micrographs illustrating transwell-migration of B16F10 cells transduced with empty vector, control or syntenin shRNA. Right: Bar graphs indicate trans-migration relative to empty vector transduced cells (taken as 100%). Note that syntenin-depleted cells always migrate more slowly than control cells. *n* = 3, bars represent mean value ± SD, n.s., non-significant, ^∗∗^*P* < 0.01, ^∗∗∗^*P* < 0.001 (Student’s *t*-test). **(C)** Confocal micrographs illustrating active integrin β1 staining (red in merge, blue corresponds to DAPI staining of the nuclei) in B16F10 cells treated with control (Ctrl) or syntenin siRNA (syntenin -), as indicated. Note the integrin β1 staining at the cell borders in controls (arrows) but not in syntenin-depleted cells. Scale bar, 10 μm.

We then evaluated the effect of syntenin silencing on the migratory potential of the various cell lines. We measured cellular migration in wound healing (**Figure 2A** and Supplementary Figure 1A) and transwell migration assays (**Figure 2B** and Supplementary Figure 1B). To exclude any influence of cell proliferation on cell migration, we performed our experiments in the presence of 1 ng/ml of mitomycin to block cell division (Dow et al., 2007). Control shRNA, used as control, did not affect syntenin migration significantly when compared to empty vector transduced cells. shRNA targeting syntenin induced a significant decrease in cellular migration by wound healing assay in B16F10 cells (down by 44% compared to control levels), in HT29 cells (down by 35 and 37% compared to control levels for the two different shRNA, respectively) and in MCF7 cells (down by 28 and 35% compared to control levels for the two different shRNA, respectively) (**Figure 2A**). shRNA targeting syntenin also induced a significant decrease in cellular migration in transwell migration assays in B16F10 cells (down by 38% compared to control levels), in HT29 cells (down by 28 and 30% compared to control levels for the two different shRNA, respectively) and in MCF7 cells (down by 35 and 36% compared to control levels for the two different shRNA, respectively) (**Figure 2B**). In an attempt to identify mechanisms that could explain perturbed migration, we compared the tubulin cytoskeleton pattern, as well as the membrane distribution of active integrin β1 in control and syntenin-depleted cells in regular 2D cultures. While tubulin cytoskeleton did not display drastic differences (data not shown), the plasma membrane/cell borders staining of the active form of integrin β1 was clearly less marked in syntenin-depleted cells than in controls (shown for B16F10 cells) (**Figure 2C**). Altogether, these results indicate that syntenin expression supports B16F10, HT29 and MCF7 cancer cell migration.

### Syntenin Loss-of-function Impairs Anchorage-independent Cell Growth and Cellular Proliferation

**FIGURE 3:**
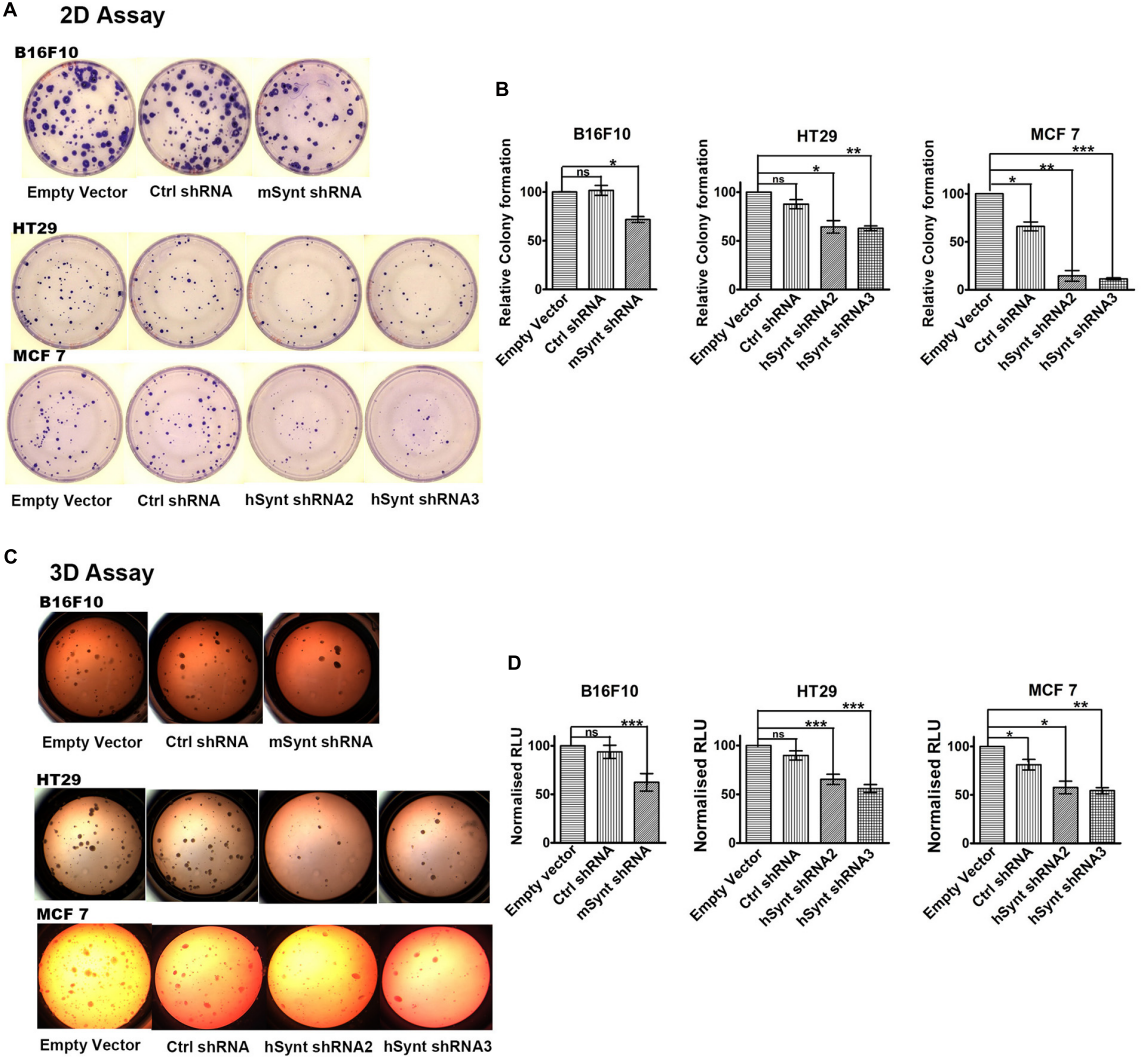
**Syntenin loss-of-function leads to reduced growth in different cancer cell models. (A)** Representative images of colony formation assays with the different cell models. **(B)** Bar graphs indicate the number of colonies formed relative to colony numbers formed by empty vector-transduced cells (taken as 100%). *n* = 3, bars represent mean value ± SD, n.s., non-significant, ^∗^*P* < 0.05, ^∗∗^*P* < 0.01, ^∗∗∗^*P* < 0.001 (Student’s *t*-test). **(C)** Representative images of soft agar colony formation assays with the different cell models. **(D)** Bar graphs indicate luciferase activities expressed in relative light units (RLUs), relative to activities measured in empty vector transduced cells (taken as 100%). Note that syntenin-depleted cells grow more slowly than the control cells. *n* = 3, bars represent mean value ± SD, n.s., non-significant, ^∗^*P* < 0.05, ^∗∗^*P* < 0.01, ^∗∗∗^*P* < 0.001 (Student’s *t*-test).

The role of syntenin in cell growth was investigated using *in vitro* plate colony formation assays (**Figures 3A,B**) and soft agar colony formation assays (**Figures 3C,D**). The number of colonies was significantly decreased in syntenin-depleted B16F10, HT29, and MCF7 cells compared to control cells, i.e., cells expressing empty vectors. In MCF7 cells, two different shRNA induced a drastic reduction of plate colony formation, decreasing, respectively, by 85 and 88% compared to control. In B16F10 and HT29 cells, the reduction of colony numbers was less drastic but still significant, with a decrease by 30% in B16F10 and by 36 or 38% in HT29 cells depending on the shRNA (**Figures 3A,B**). We also performed soft agar colony formation assays to test cellular abilities to form colonies in three dimensions because this assay is considered as the most stringent test for the detection of anchorage-independent tumor cell growth (Horibata et al., 2015). shRNA targeting syntenin induced a 49% decrease in the growth of B16F10 cells, a 20 or 33% decrease in the growth of HT29 cells and a 44 or 46% decrease in the growth of MCF7 cells (respective values for the two different shRNA) (**Figures 3C,D**). Control shRNA, used as control, did not significantly affect colony formation, neither in plate nor in soft agar assays, when compared to empty vector transduced B16F10 and HT29 cells. Surprisingly, control shRNA significantly affected colony formation of MCF7 cells in both assays. The reason for this specific effect in MCF7 cells is unknown. While *a priori* this observation does not change the conclusion that syntenin depletion affects the growth of MCF7 cells, it indicates that from a quantitative point of view, data should be interpreted with caution.

**FIGURE 4:**
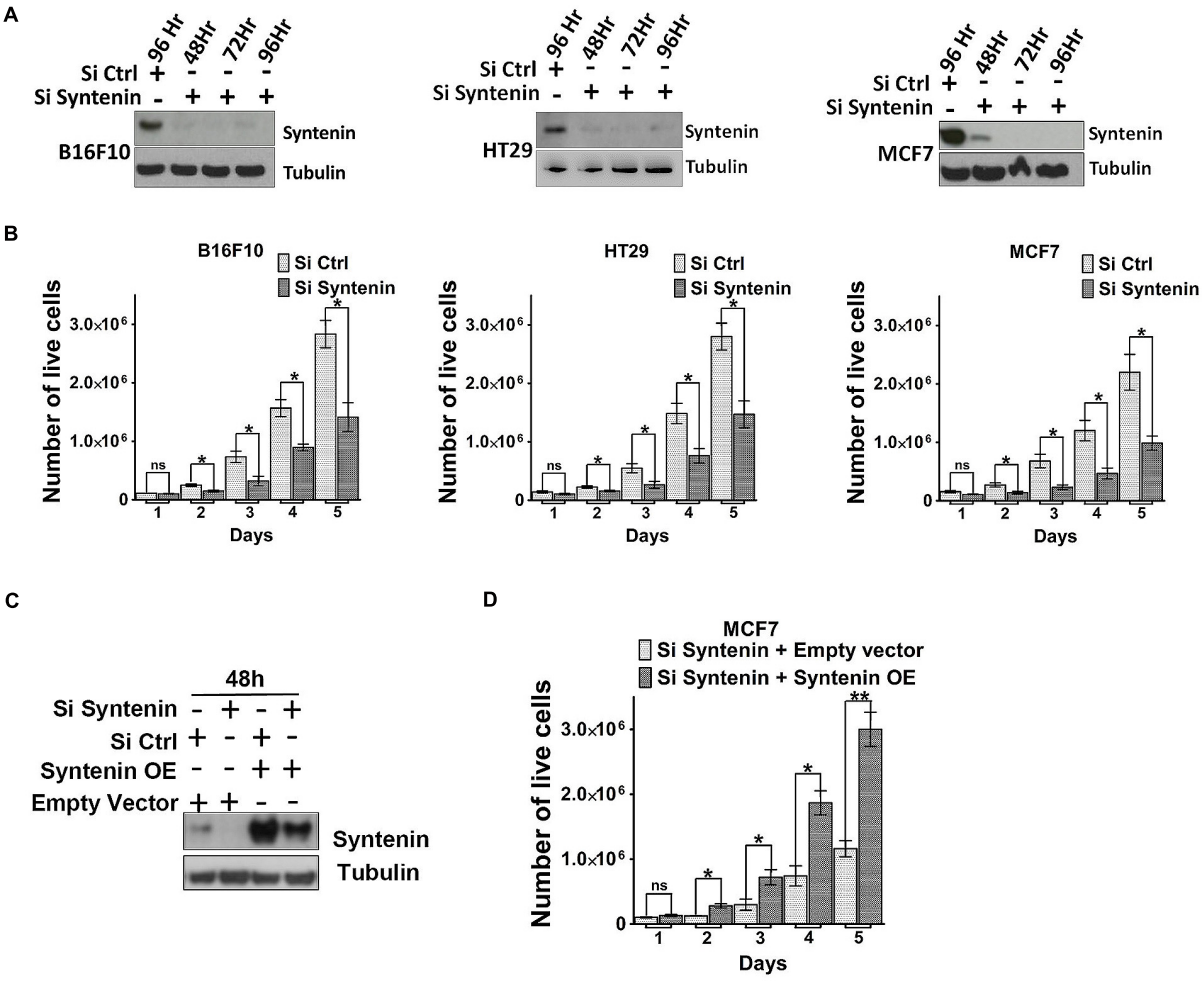
**Syntenin loss-of-function leads to reduced proliferation in different cancer cell models. (A)** Western blots illustrating syntenin expression levels, at different time point, in B16F10, HT29, and MCF7 cells, after transfection with non-targeting (Si Ctrl) or syntenin (Si Syntenin) siRNAs. Tubulin was used as a loading control. **(B)** Bar graphs indicate the absolute number of living cells measured after different days of culture, as indicated. Note that significant differences were already observed at day 2. *n* = 3, bars represent mean value ± SD, n.s., non-significant, ^∗^*P* < 0.05 (Student’s *t*-test). **(C)** Western blots illustrating Syntenin expression levels in MCF7 cells 48 h after transfection with different constructs, as indicated. SiRNA Syntenin (Si Syntenin); non-targeting siRNA (Si Ctrl); expression vector for human Syntenin non-tagged and mutated for the siRNA targeting sequence (Syntenin OE); empty expression vector (Empty vector). Tubulin was used as loading control. **(D)** Bar graph indicating the absolute number of living MCF7 cells in Syntenin rescue experiments after different days in culture. *n* = 3, bars represent mean value ± SD, n.s., non-significant, ^∗^*P* < 0.05, ^∗∗^*P* < 0.01 (Student’s *t*-test).

We also tested the effect of syntenin depletion by transiently downregulating syntenin expression, using small interfering RNA (siRNA). Effective syntenin knockdown by mouse and human syntenin siRNAs over a period of 4 days was validated by Western blot analysis (**Figure 4A**). In these experiments, syntenin expression was downregulated by 90–95% compared to controls. We tested the proliferation of B16F10, HT29, and MCF7 cells transiently transfected with non-targeting control siRNA (si Ctrl) and syntenin siRNA (si Syntenin) by counting the number of viable cells every day over a period of 5 days (**Figure 4B**). Control B16F10 and HT29 cells (si Ctrl) showed a 30-fold increase in cell number after 5 days, while control MCF7 cells showed a 20-fold increase. Syntenin-depleted B16F10 and HT29 cells (siSyntenin) showed a 15-fold increase in cell number after 5 days, while syntenin-depleted MCF7 cells showed a 10-fold increase. A significant difference between controls and syntenin-depleted cells was observed in all models at day 2 and later times (**Figure 4B**). An effect of syntenin depletion on cell death could be ruled out because we observed in controls and syntenin depleted cells a similar extremely low number of Trypan blue (Supplementary Figure 2) and annexin-V positive cells (Supplementary Figure 3). To further validate syntenin effects on cellular proliferation, we also performed rescue experiments with MCF7 cells. Cells were treated with control (si Ctrl) and syntenin siRNAs (si Syntenin) for 24 h and then transiently transfected with an expression vector for wild-type syntenin mutated for the siRNA targeting sequence (syntenin OE) or the empty vector as a control. Total cell lysates analyzed by Western blot indicated rescue of syntenin expression above the control levels in MCF7 cells at 48h (**Figure 4C**), but these levels were still in the physiological range commonly observed in cell cultures. Rescued MCF7 cells showed a significant improvement of proliferation at day 2 and later, and 30-fold increase in cell number after 5 days of culture (**Figure 4D**). We assume that this increase in proliferation (by a factor 1.5 at day 5 when compared to control cells-Si Ctrl in **Figure 4B**), might result from the slight gain of syntenin expression in rescued cells. Altogether, the above data indicate that syntenin supports the capacity of single cells to form colonies, anchorage-independent cell growth, and proliferation in B16F10, HT29, and MCF7 cells and that cellular proliferation might be directly correlated to syntenin expression levels.

### Syntenin Loss of Function Impairs G1/S Cell Cycle Transition

**FIGURE 5:**
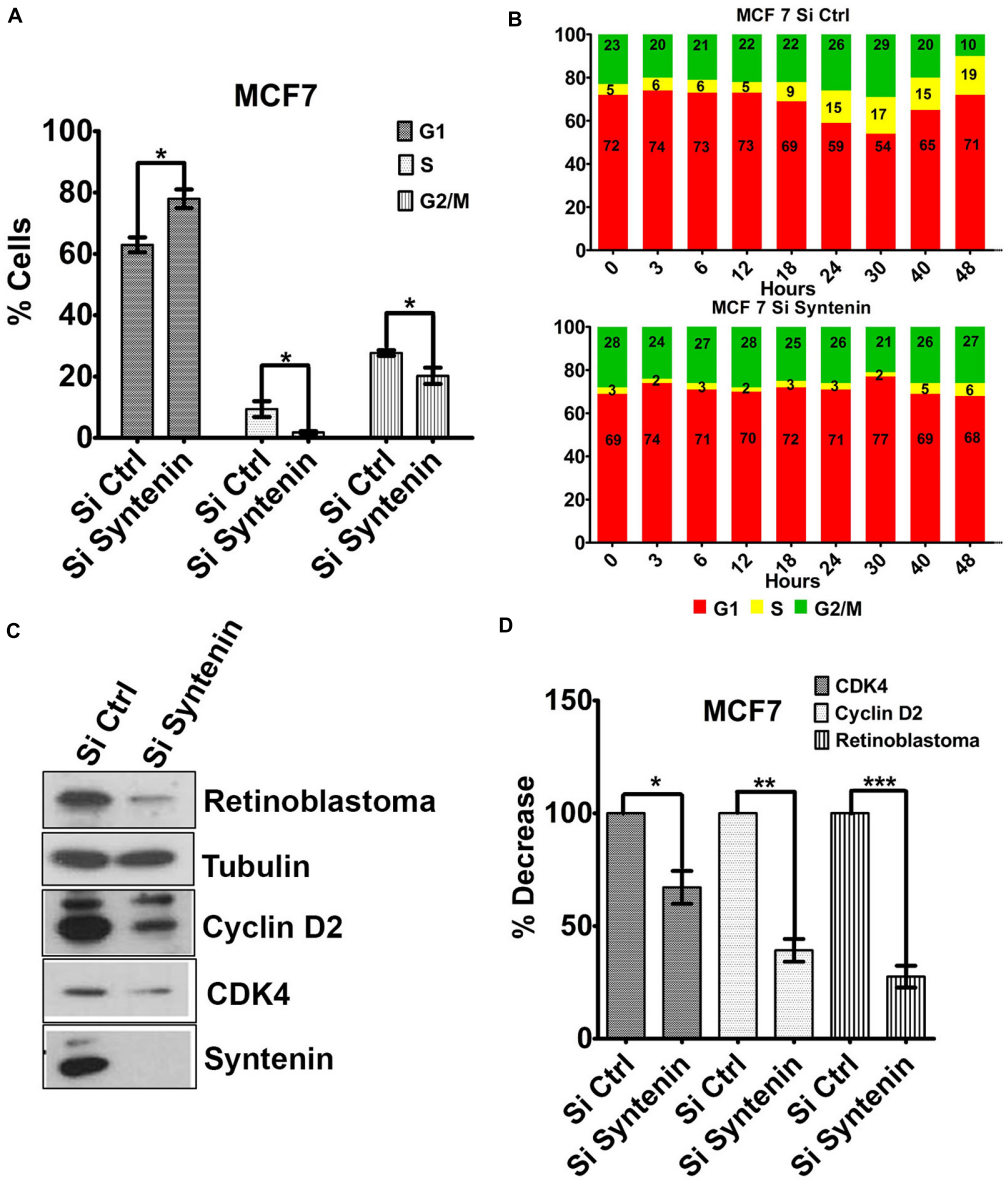
**Downregulation of syntenin impairs cell cycle G1/S transition in MCF7 cells. (A)** Bar graph representing the percentage of cells in G1, S, and G2/M cell cycle phases after synchronization. Cells were transfected with non-targeting siRNA (Si Ctrl) or Syntenin siRNA (Si Syntenin). *n* = 5, bars represent mean value ± SD, ^∗^*P* < 0.05 (Student’s *t*-test). **(B)** Bar graphs illustrate one kinetic experiment indicating the percentage of cells in G1, S, and G2/M cell cycle phases after serum stimulation, at different time points, as indicated, in cells transfected with non-targeting siRNA (top) or Syntenin si RNA (bottom). **(C)** Western blot comparing the expression levels of different cell cycle markers, as indicated, in control cells (Si Ctrl) and Syntenin-depleted (si Syntenin) cells. Tubulin was used as loading control. Note that the expression of Cyclin D2, CDK4, and Rb is downregulated in syntenin-depleted cells. **(D)** Bar graph indicating the expression level of cell cycle markers in Syntenin-depleted cells relative to controls (taken as 100%). *n* = 3, 4, and 5 for Cyclin D2, Rb, and CDK4, respectively, bars represent mean value ± SD, ^∗^*P* < 0.05, ^∗∗^*P* < 0.01, ^∗∗∗^*P* < 0.001 (Student’s *t*-test).

To better understand the effect of syntenin loss-of-function on cell growth and proliferation, we tested whether cells were arrested in a particular phase of the cell cycle. Syntenin-depleted (si Syntenin) and control (si Ctrl) MCF7 cells were synchronized by serum starvation for 24 h. Cells were then serum-stimulated for 24 h and analyzed for different cell cycle phases by flow cytometry. Syntenin-depleted cells showed a significant increase in G1 phase and a significant decrease in S and G2/M phases when compared to control cells (**Figure 5A**). The effects of serum-stimulation on the different phases of the cell cycle were also analyzed at different time points over a period of 2 days in two independent experiments; see **Figure 5B** for one illustration. At early time points (from 0 to 12 h), control and syntenin-depleted cells did not show drastic differences in G1 phase (on average 73% in controls and 71% in depleted cells) but the percentage of S phase in control cells was twice more important compared to syntenin-depleted cells (on average 5.5% in controls and 2.5% in depleted cells). Starting from 18 h, S phase was increased to reach 19% at 48 h in control cells, while in syntenin-depleted cells, S phase poorly increased over time to reach at maximum 6% after 48h of serum stimulation. Moreover, the ratio of cells in G1, S, and G2/M stayed quite constant all along the experiment in syntenin depletion conditions on the contrary to what we observed in controls (**Figure 5B**). Taken together, these data suggest that syntenin depletion induces a defect in G1/S cell cycle transition. Of prime importance in this process is cyclin D2, which binds and activates cyclin-dependent kinases 4 (CDK4), thereby phosphorylating Retinoblastoma protein (Rb) and promoting progression from mid to late G1 (Sherr, 1994). We therefore tested for the expression levels of these three cell cycle regulators of G1/S transition by Western blot and observed that they are all significantly downregulated in syntenin-depleted cells compared to control cells (**Figures 5C,D**). Altogether, our data indicate that syntenin might control cell growth by acting on G1/S cell cycle transition.

## Discussion

Distinct assays and different cancer cell types (melanoma, colon and breast) from various origins (mouse and human) were used in this study to investigate the effects of syntenin knockdown. All assays showed that syntenin depletion significantly decreases tumor cell migration, growth, and proliferation. Our results with the commonly used B16F10 cells corroborate previously reported studies with other mouse melanoma models showing that syntenin overexpression promotes melanoma invasion, motility and anchorage-independent growth (Boukerche et al., 2005). Additionally, our experiments in human colon HT29 cells, show that depletion of syntenin reduces cellular migration, growth and proliferation. This is consistent with a study from Lee et al. (2011) showing that syntenin gain of function stimulates the migration of HT29 cells, and other colon cancer cells in transwell assays, while syntenin knockdown by siRNAs have the opposite effects (Lee et al., 2011). Although not directly tested in HT29, the same study also illustrated a role for syndecan-2-syntenin interaction in the migratory potential of colon cancer cells, an observation directly in favor of our hypothesis that syntenin effects in cancer cells are primarily due to its effects on syndecan metabolism. Our results with the MCF7 breast cancer cells corroborate pioneer data from Koo et al. (2002) and more recent data by Yang et al. (2013). Indeed, Koo et al. (2002) identified syntenin by differential gene expression profile as a metastasis-related gene in breast cancer cells. They showed that gain of syntenin expression in MCF7 cells stimulates their migration and invasion in transwell assays. Moreover they identified syntenin PDZ2 domain, the syndecan high-affinity interacting domain of syntenin (Grootjans et al., 1997, 2000), to be responsible for the migratory stimulating effect. By studying a larger panel of breast cancer cells, Yang et al. (2013) found that syntenin expression levels correlate with the metastatic potential of these cells. Using the MDA-MB-231 breast cancer cell model, they revealed the impact of syntenin gain- and loss-of-function on the migratory and invasive behavior of these cells. Finally, using xenografts, they showed that syntenin overexpression promotes tumor growth and lung metastasis *in vivo* (Yang et al., 2013).

Consistent with the low migratory rate, the data presented here show that syntenin-depleted cells present a defect in the plasma membrane localization of active β1-integrin. This observation is totally in line with previous data showing that, by allowing syndecan recycling, syntenin might support HS-dependent signaling molecules to be present and active at the plasma membrane (Zimmermann et al., 2005) and that, integrin trafficking is required for cellular adhesion and migration (Humphries et al., 2015). Integrin related or not, we observed a decrease in the percentage of cells in the most proliferative S and G2/M phases and a defect in the cell cycle G1/S transition phase in syntenin-depleted cells. We also documented that the growth regulatory molecules and G1/S key regulators, Cyclin D2 and CDK4 (Sherr, 1994) are downregulated upon syntenin inhibition. Alltogether, these observations are in line with a role for syntenin in tumor progression. Moreover, Cyclin D1 and CDK4 downregulation through syntenin depletion was previously reported in head and neck squamous cell carcinoma (Oyesanya et al., 2014). Cyclins D are synthesized as long as growth factor stimulation persists. Their destruction, in response to growth factors deprivation for example, results in the failure of cells to enter S phase. A potential explanation for the effects of syntenin depletion on Cyclins D and CDK4 would be that syndecan associated growth factor receptor systems such as FGF-2-FGFR1, are directed toward degradation instead of being recycled to the plasma membrane (Zimmermann et al., 2005). In this manner, syntenin-depleted cells would be less able to respond to serum stimulation and thus less able to stimulate Cyclins D synthesis and entry into the S phase. Yet, there is also a possible more direct explanation. Indeed, Cyclin D1 and Cyclin D2 present a class II PDZ-binding motif (PDZBM) at their C-terminal domain (DVDI and DIDL, respectively). A direct interaction between syntenin PDZ domains and Cyclins D PDZBM could also control G1/S transition as syntenin has also been detected in the nucleus (Zimmermann et al., 2001).

In agreement with our results in MCF7 cells, a study from Qian et al. (2013) showed that syntenin promotes MDA-MB-231 breast cancer cells G1/S transition phase (Qian et al., 2013). Interestingly, the authors also propose that syntenin levels in breast cancer cells is inversely correlated to estrogen receptor levels and propose that syntenin maintains the growth of breast cancer cells when estrogen-signaling pathway is not available. Although we similarly observed the negative correlation between syntenin levels and estrogen-receptor status (data not shown), this would not fit our hypothesis that syntenin effects in cancer are supported by its effect on syndecan trafficking. More importantly, our results with estrogen receptor-positive MCF7 cells demonstrate that even low levels of syntenin are sufficient to modulate cancer cell behavior.

## Conclusion

Exploration of syntenin as a pharmacological target to inhibit cancer progression is probably a valuable objective with potential broad impact.

## Author Contributions

RK, BR., FL, JF, AC, AR, RG, carried out the molecular, biochemical, and cell biological work; RG, PZ supervised the research and wrote the manuscript; all authors were invited to revise the manuscript.

## Conflict of Interest Statement

The authors declare that the research was conducted in the absence of any commercial or financial relationships that could be construed as a potential conflict of interest.
