# Supplementary material for: Syntenin controls migration, growth, proliferation, and cell cycle progression in cancer cells
Source: Front Pharmacol. 2015 Oct 21;6:241. doi: 10.3389/fphar.2015.00241 (PMC4612656; doi:10.3389/fphar.2015.00241)
Supplement: Supplementary file 1 [file Image_1.PDF]

Supplementary figure. 1

(A)

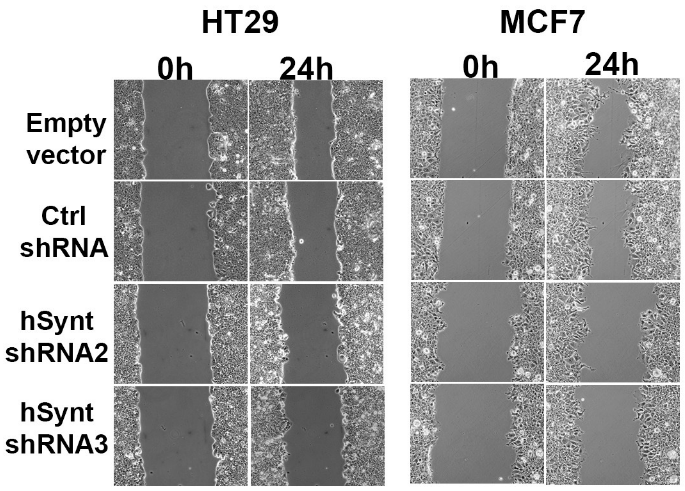

(B)

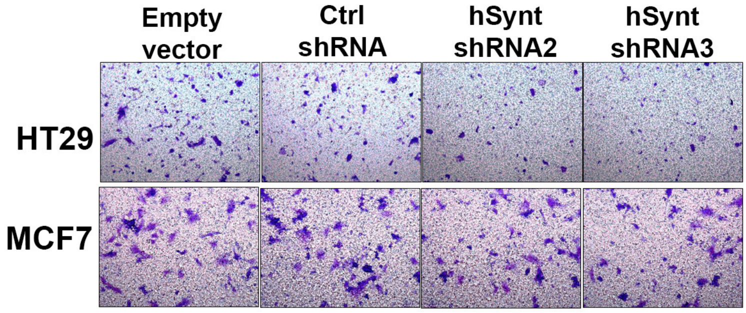

Supplementary figure. 2

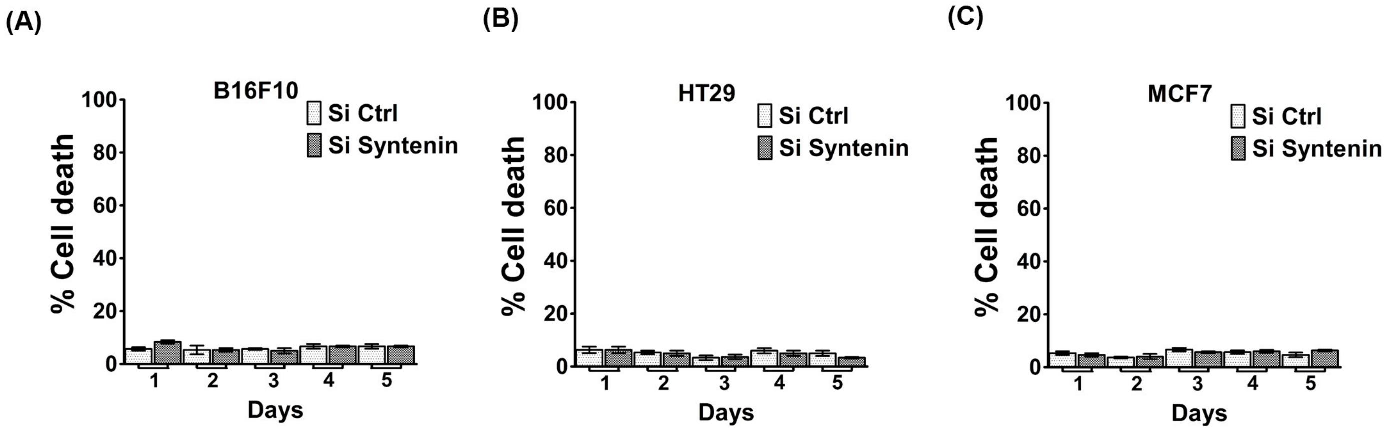

Supplementary figure. 3

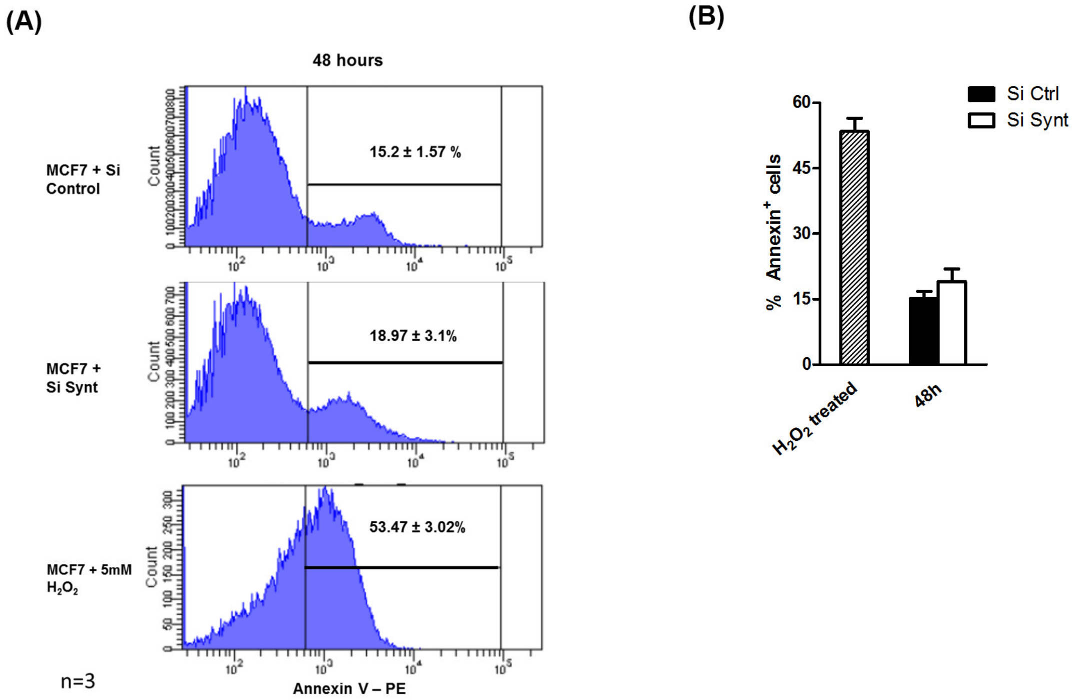

Supplementary figure 1. Syntenin loss-of-function reduces the migration of human cancer cells.

(A) Phase-contrast micrographs illustrating migration by wound healing of HT29 and MCF7 cells monolayers transduced with empty vector, control or syntenin shRNAs expression vectors. Images were taken at different time points after wounding, as indicated. **(B)** Micrographs illustrating transwell migration of HT29 and MCF7 cells transduced with empty vector, control or syntenin shRNAs expression vectors.

Supplementary figure 2. Effect of Syntenin si RNA on cell death in different cancer cell models.

B16F10, HT29 and MCF7 cells **(A, B and C respectively),** after transfection with non-targeting (Si Ctrl) or syntenin (Si Syntenin) siRNAs. The percentage of dead cells (colored with Trypan blue) was measured after different days of culture.

Supplementary figure 3. Evaluation of the percentage of apoptotic cells by annexin-V staining. MCF7 cells transfected with non-targeting (si Ctrl) or syntenin (si Synt) siRNAs were collected 48hours after transfection and subjected to annexin V binding assay as per the manufacturer’s protocol (BD Biosciences). The stained cells were analyzed by Flow cytometry. Untransfected cells were incubated with 5mM hydrogen peroxide (H2O2) for 90min prior to annexin V binding as a positive control for the assay. The experiment was performed in triplicates**.** **(A)** Flow cytometric analysis of annexin V-PE staining. Representative histograms of 3 independent experiments with similar results**.** **(B)** Bar graphs representing the percentages of annexin V-positive cells in the H2O2-treated, the Si Ctrl- and the Si Synt-transfected MCF7 cells. Data are mean ± SEM.
